# Decisions regarding antibiotic prescribing for acute sinusitis in Norwegian general practice. A qualitative focus group study

**DOI:** 10.1080/02813432.2023.2274328

**Published:** 2023-11-29

**Authors:** Jorunn Thaulow, Torunn Bjerve Eide, Sigurd Høye, Holgeir Skjeie

**Affiliations:** Department of General Practice, Institute of Health and Society, Antibiotic Centre for Primary Care, University of Oslo, Oslo, Norway

**Keywords:** Sinusitis, qualitative research, focus group, general practice, antibiotics, guidelines, decision making

## Abstract

**Background:**

Acute sinusitis is a frequent reason for primary care visits. Most patients recover within two weeks without antibiotic treatment. Despite this, about 50% of patients with acute sinusitis in Norwegian general practice are still prescribed antibiotics. We do not know the reason behind this discrepancy.

**Aim:**

To explore the clinical decision-making process and reasons for treatment with antibiotics for acute sinusitis among Norwegian general practitioners (GPs).

**Methods:**

Five focus group interviews were conducted (*N* = 25) in different parts of Norway, including GPs of various age, gender, and experience. The interviews were analysed using Systematic Text Condensation.

**Results:**

The results showed a very diverse management of acute sinusitis among GPs, with decisions regarding antibiotics not always aligning with guideline recommendations. Many of the GPs did not agree with the Norwegian guidelines for antibiotics and chose something other than phenoxymethylpenicillin as their first choice. Clinical predictors emphasized in decision-making were pain complaints and patient exhaustion. Pragmatic factors such as weekday, travel plans, or a full waiting room could also influence the decision.

**Conclusion:**

GPs found it difficult to identify when patients would benefit from antibiotic treatment for acute sinusitis, and different strategies were used to make prescribing decisions. For several GPs the degree of pain was one of the decisive reasons for antibiotic prescribing, however the guidelines for antibiotics do not give sufficient advice regarding pain treatment. These results suggest a need for revaluation of guideline contents and the way they are communicated to GPs.

## Key Points

- We know that antibiotics are often overused in the treatment of acute sinusitis, but there is lack of knowledge about the reasons behind GPs decision to prescribe them.
- There were considerable variations in clinical decision-making regarding the treatment of acute sinusitis among Norwegian GPs.
- The patients’ pain level, as well as pragmatic factors, were important deciding factors in the prescription of antibiotics.
- Guidelines for treatment regarding acute sinusitis may include more emphasis on pain management.

## Introduction

The challenge of combating antimicrobial resistance (AMR) is increasing worldwide. AMR leads to prolonged illness, higher healthcare expenditures, and even deaths. In Europe, an estimated 23,000 annual deaths are attributes to multiresistant infections [1]. Hence, proper evaluation of the need for antibiotic treatment for potential bacterial infections is important in the context of the global crisis of antibiotic resistance [2]. Acute sinusitis (AS) has a very high incidence worldwide and is hence a common reason for encounters in primary care. AS is usually the consequence of a viral common cold, with approximately 0.5–2.0% progressing to secondary bacterial infection [2]. The condition is usually self-limiting, although serious complications that can lead to life-threatening situations have been reported. AS is also commonly treated with antibiotics in primary care, although most patients do not benefit from antibiotics [3,4].

A systematic review study from 2016 showed that approximately 85% of patients with suspected sinusitis will recover after 7–15 days without antibiotic treatment [5]. A recently published Cochrane report concludes that antibiotics have no place in treating uncomplicated AS, regardless of etiology, since the potential benefit of antibiotic therapy is marginal and must be seen in the context of risk of side effects and resistance development [6]. The National guidelines for antibiotics in primary care recommend withholding antibiotics for patients with mild to moderate symptoms of sinusitis, and to offer antibiotics only to patients with pronounced symptoms and purulent nasal discharge lasting for more than one week’s duration [7]. Phenoxymethylpenicillin is the recommended first-line antibiotic. This is largely in accordance with recommendations and guidelines in the other Scandinavian countries [8,9].

In studies from England and the United States of America, as many as 84–91% of patients with AS received antibiotics [10,11]. A study from Danish general practice in 2011 found that around 70% were prescribed antibiotics for AS [12]. A recent Norwegian study found that antibiotics are prescribed to 49% of patients with acute sinusitis in Norwegian general practice. This shows a trend toward more restricted use, and is an expected reduction after a decade of campaigns to combat antibiotic resistance [4]. Despite this effect, too many patients with AS are still treated with antibiotics. It is well known that the diagnosis of sinusitis is difficult, and the clinical uncertainty is a problem [5]. In one study, pain in the sinus cavities was detected in 95% of patients who presented with suspected AS. Pus or mucus upon antral puncture was found in only 53% of patients where the GP suspected sinusitis [12]. In a newly published systematic review, the authors found that pain in the teeth, purulent nasal discharge, and elevated CRP were the best predictors for culture-confirmed bacterial sinusitis [13]. Several studies point out that many AS patients have migraine or other types of headaches and are misdiagnosed with sinusitis [14,15]. The downstream effect of the cytokine cascade initiated in migraine physiology can also cause rhinologic symptoms, including rhinorrhea, congestion and lacrimation [15]. In some cases, this can lead to incorrect use of antibiotics before realizing the true etiology of the symptoms [16].

Several studies have discussed reasons for prescribing antibiotics that are at odds with current guidelines [17]. GPs’ ingrained habits regarding antibiotic prescribing are crucial for whether a patient will receive treatment with antibiotics for sinusitis-like symptoms [18,19]. Cars and Håkansson concluded that ‘Doctors have an individual and very constant pattern of prescribing antibiotics, and it seems that the diagnoses are often given to justify the treatment, rather than the other way around’ [18]. A Swedish study suggests that if the doctor spends more time listening to the patient, they may reduce the prescription of antibiotics without reducing patient satisfaction [19]. Another study found that teaching all staff in the GP office correct use of antibiotics was important when looking at factors associated with low antibiotic prescribing for respiratory tract infections [20].

To our knowledge, there are so far no qualitative studies exploring the reasoning behind antibiotic prescriptions for AS in primary care. The aim of this study is to obtain knowledge of the factors that come into play when GPs decide whether to prescribe antibiotics to patients with symptoms of AS.

## Materials and methods

### Data collection

We performed a qualitative focus-group study. When recruiting focus groups, we aimed for a variation in experience, age, and gender, as well as representation from different regions of Norway. The participants in three of the groups were part of a Continuing Medical Education (CME) group that would meet regularly. Two of the groups were made up of colleagues in a GP practice. Four of the focus groups were recruited through networks from three of the four authors. The final group was recruited through PraksisNett, a Norwegian general practice national research network. Each focus group met once for 60–90 minutes, and the interviews were recorded digitally. JT acted as the moderator for all five focus groups. HS and SH participated in one each of the first and second groups. The last three focus groups were conducted by JT alone. Two of the groups met at the participants’ practices, while three groups met at the home of GP. In all groups, the GPs were familiar with each other before the study.

We used a semi-structured interview guide with open ended questions. We formulated the questions based on a literature review and the authors’ experiences regarding antibiotic prescribing in primary healthcare. We have included the interview guide as an appendix. The guide was adjusted after each focus group. During the interview period it changed from thoughts regarding the general treatment of sinusitis to a more focused guide concerning the deliberations on when antibiotics should be prescribed. To determine when we had sufficient information, we used Malterud’s concept of information power as a method to ascertain adequate internal validity [21]. We have provided further elaboration of this in the methodology discussion section.

### Analysis

JT, SH, and HS designed the protocol and initial interview guide. JT transcribed all the interviews verbatim. JT, HS and TBE analyzed the transcripts using systematic text condensation (STC) as described by Malterud [22]. Systematic text condensation is a descriptive and exploratory approach used for conducting thematic cross-case analysis of qualitative data. The process involves the following stages: (1) total impression – from chaos to themes; (2) identifying and sorting meaning units – from themes to codes; (3) condensation – from code to meaning; (4) synthesizing – from condensation to descriptions and concepts [22]. We started by reading all the interviews to get an overall impression and identify preliminary themes before the first meeting. Based on these themes, we began the coding process together. We selected meaning units for each code group describing the GPs’ thoughts regarding the use of antibiotics for acute sinusitis. TBE independently read the transcripts and identified meaning units before the first meeting with the whole team. As part of the process, we made a clarifying code-tree together. All authors agreed on the final code groups, subgroups, and illustrative quotes. We made condensates illustrating the contents of each code group, and finally, we performed the last step of STC, synthesizing the condensates to present a reconceptualized description of the GPs’ thoughts on sinusitis and when antibiotics are necessary. We used a stepwise approach, conducting a preliminary analysis after the first interviews, allowing for adjustments to the interview guide and objectives of the interviews. JT is an ear nose and throat (ENT) specialist, HS and TBE are specialists in family medicine. SH is a general practitioner, leader of the Antibiotic Centre for Primary Care and medical editor of the National guidelines for antibiotics in primary care. All four authors are researchers at The Department of General Practice, University of Oslo, and are associated with the Antibiotic Centre for Primary Care (ASP), a national center of competence, placed under The Department of General Practice. Our study is part of the BASIC (Better treatment for Acute Sinusitis In primary health Care) project.

The quotes were translated from Norwegian to English by the authors. We used NVivo12 software to analyze the data. The Consolidated criteria for Reporting Qualitative studies (COREQ) were used in the reporting of the data.

## Ethics

The Norwegian Centre for Research Data approved data protection (ref.no. 266839). Since the study did not involve any sensitive patient information or interventions (ref.no. 98690), it did not require approval from the Regional Committee for Medical and Health Research Ethics. The informants provided informed consent to participate in the study. To maintain confidentiality during the focus group discussions, sensitive information was redacted from the interviews when transcribed, and non-identifiable designations were used when quoting. After the analysis was completed, we conducted informant validation with three participants from three different focus groups. We presented the main findings and welcomed comments.

## Results

From February 2020 to May 2022, we interviewed five groups of GPs from four different regions of Norway. The focus groups were conducted in one large city (approximately 700,000 inhabitants), two large towns (100–200,000 inhabitants) and one smaller town (approximately 20,000 inhabitants). Two of the focus groups consisted of three participants, while the rest of the groups contained six, seven and five participants. Table 1 presents the characteristics of the participants.

**Table 1.** Characteristics of the participating GPs.

Total number of participants	25
Median age (range)	42 (27–58)
Women	20
Men	5
Median years (range) of GP experience	11.3 (0.4–26)
Specialist in general practice	18

In a reflexive analyzing process, we reached a common understanding of three main themes regarding GPs’ decisions on antibiotic treatment for AS:

- Guidelines are known but not always followed.
- Pragmatic factors influence the decision to prescribe antibiotics.
- Pain and exhaustion are treated with antibiotics.

### Guidelines are known but not always followed

All participants acknowledged the importance of guidelines and the need for a reduction in the use of antibiotics. However, there were varying attitudes among the participants regarding which patients should be treated with antibiotics and when non-antibiotic treatment should be recommended. The majority indicated that if the general condition was good and the infection had not lasted too long, they would initially recommend non-antibiotic treatment.

Most of the GPs did not spontaneously mention the guidelines when discussing how they chose to treat sinusitis. When asked directly, some admitted that they did not regularly consult the guideline recommendations but claimed to have general knowledge of their content. However, several participants expressed disagreement with the guideline recommendations and preferred to follow their own way approach to treatment. Clinical judgment was identified as the most important decision-making tool.

Many of the GPs indicated that if they decided to prescribe antibiotics, their first choice would be phenoxymethylpenicillin, which is in line with Norwegian guidelines. However, quite a few GPs shared their experiences that phenoxymethylpenicillin was ineffective for treating sinusitis. Some had encountered patients who did not improve with phenoxymethylpenicillin, while others explained that sinuses are a closed system without blood vessels, which is why phenoxymethylpenicillin would not be effective.

My opinion is that treatment with phenoxymethylpenicillin is practically ineffective. I may be wrong here, but that is what I have argued for the patient, and they obviously do not want medicine that does not work. (Man 33, group 5)

In line with this, some GPs’ chose to prescribe an alternative antibiotic from the beginning to avoid switching later. Several participants mentioned that if patients reported good results with one type of antibiotic in previous episodes of sinusitis, it was difficult to deny their request for the same antibiotic, even if it went against the guidelines.

A: If you decide to prescribe antibiotics, maybe you should consider clindamycin or ciprofloxacin (Man 44, group 5)

B: But that is not according to the guidelines? (Woman 42, group 5)

A: No, but that is how I understand antibiotics in terms of tissue penetration. Otherwise, you would have to switch to a high dose of regular phenoxymethylpenicillin, …. That’s perhaps why I am hesitant to prescribe phenoxymethylpenicillin, I can′t understand how it can have any effect. (Man 44, group 5)

Many of the participants found it difficult to determine whether a sinusitis was caused by a virus or bacteria. This led to uncertainty related to the need for antibiotics, which was a decisive factor for many when choosing a treatment approach.

I can hardly remember anyone having an effect of phenoxymethylpenicillin for sinusitis, but it could be because it is viral, I suppose. (Woman 42, group 3)

### Pragmatic factors influencing decision-making

#### Sympathy with the patient

Some of the doctors had suffered from sinusitis themselves. They emphasized how painful it was and that they sympathized with patients who wanted to try antibiotics. In some cases, feelings of empathy overrode guideline recommendations when deciding on antibiotic treatment.

It’s really painful to have a proper sinusitis, it hurts a lot, so I do understand that you want to try something. If the patient manages to convey enough suffering, I may give in a bit, I feel so sorry for that patient that I go: OK then! (Woman 57, group 5)

Several participants highlighted how sinusitis could cause fatigue. If the patient appeared to have a reduced general condition, this often strengthened the indication for antibiotics. One participant discussed which patients ‘deserved’ antibiotics and indicated that if they seemed very affected by pain, they deserved antibiotics. Some participants also stated that the patient′s life circumstances could affect their decision, for example if the patient seemed very tired and all the children were sick.

#### Contextual factors

In the interviews, practical factors were frequently mentioned as influential when making decisions regarding antibiotic prescriptions. One such factor was day of the week.

I do think I prescribe antibiotics more easily on Fridays than on Mondays (laughter). When I work in the out-of-hours service, like Friday after lunch; I think everyone gets what they want from me. (Woman 44, group 3)

Time pressure and a full waiting room were also factors that could lower the threshold for antibiotic prescription to this patient group. Several doctors also indicated that they prescribed antibiotics more easily if the patient was planning to travel in the next few days.

#### The doctor-patient relationship

Many of the doctors reflected on their relationship with patients as their primary care physicians and believed that knowing the patients made it easier to understand and interpret their symptom presentations. The importance of how the patient expressed their level of suffering and how the doctor perceived the symptoms was highlighted by several participants as crucial in determining the choice of treatment. The doctor-patient relationship could influence the prescription tendencies, as it could lead to more conservative prescribing practices or giving in to patient pressure. Some participants expressed concerns over an increase in workload for GPs and feared that reduced continuity of care could result in higher antibiotic usage. They drew from their experiences in the out-of-hours medical service, where they had a lower threshold for prescribing antibiotics to unfamiliar patients.

Again, it comes down to knowing the patients. Some patients have insane pain every time, you know, while others do not typically have such presentations. So, it is clear that knowing the patients and their pain presentation, is very helpful. (Woman 38, group 2)

The patients’ previous experiences were also deemed significant. Some patients had strong opinions about the type of medication they needed to recover and knew exactly how to make the doctor listen to them. A few patients were described as demanding or pushy. This could challenge the GP’s patience and result in prescriptions against the doctor’s own convictions, simply to conclude the consultation.

If the question is whether you have done something you did not really want to do, because you were pressured to do so, then the answer is yes. There are some patients who are just a thorn in your side you know, and they never give up. They bother the secretaries too, and they will return again and again until they get what they want. (Woman 43, group 1)

A few GPs also mentioned that if the patient was a family member, or a colleague, or if the GPs were treating themselves, this could lower the threshold for prescribing antibiotics at an early stage.

### Antibiotics for pain and exhaustion

#### Patient’s appearance

Several GPs emphasized that the appearance of fatigue and exhaustion in patients with sinusitis could serve as a crucial factor in their decision-making process. Several stated that if the patient appeared tired and fatigued, it could be a decisive factor when considering antibiotic treatment. The overall clinical impression was described as crucial, and some claimed that they could often immediately determine from the patient’s appearance what kind of treatment would be necessary. Sinusitis patients who required antibiotics were described as pale, fatigued and in obvious pain.

I think it’s very easy to see it from their appearance. I don’t exactly know what it is, dull eyes, they are obviously in pain, and you can hear it in their voice too. (Woman 44, group 3)

#### Patient’s pain

The patient’s pain was consistently emphasized when the doctors described sinusitis patients in the interviews, with several stating that you could assess the level of pain just by looking at the patient. Headache or pain in the midface was often a central part of why patients felt they could not work or were too weary to continue without antibiotic treatment. Many GPs regarded the degree of pain as critical in deciding whether to prescribe antibiotics.

True sinusitis, which I have gained some respect for, is indeed painful. You will meet a patient with quite a high level of pain. (Woman 58, group 4)

Some participants reflected that the pain is typically the reason why patients see the GP, and if the patients seemed significantly affected, they found it reasonable to prescribe antibiotics to make them feel better.

In my opinion it’s similar to otitis, where pain is still an indication for antibiotics. You should mostly not use antibiotics, but if you are planning to prescribe them, pain is one of the criteria. I think it is entirely reasonable to use that as a criterion. It may not work, but you just have to give them the best medication available. (Woman 56, group 4)

#### Fever and CRP

Several doctors interpreted the presence of fever as an indication of a higher likelihood of bacterial origin for the infection. Fever in combination with reduced general health condition in patients with AS was seen by many GPs seen as a good indication for treatment with antibiotics.

There were divergent opinions regarding the use of CRP. About half of those who expressed an opinion thought it was a useful tool, for both reassuring patients that they did not need antibiotics and enabling the GP to rule out bacterial etiology.

CRP rarely shows anything, but if it is high and symptoms clearly tell me it is sinusitis, those are some of the few times I consider using antibiotics. (Woman 57, group 2)

The other half thought the test was redundant because an elevated CRP was perceived as uncommon in sinusitis. They viewed sinusitis as a clinical diagnosis and believed there was no guarantee for elevated CRP even with a bacterial infection.

## Discussion

### Summary of main results

The clinical considerations that precede the decision to prescribe or not prescribe antibiotics for AS varied among the GPs. However, it was a common goal to provide high-quality, and compassionate health care and to use antibiotics appropriately. We found that the patients’ report of pain and pressure from the sinuses were symptoms that many doctors deemed crucial in the decision of whether or not to treat with antibiotics. Some doctors emphasized the importance of following the official recommendations. However, many did not use the guidelines, or chose not to follow them because they did not agree with the recommendations.

#### Comparison with existing literature

We found that the pain level in patients were a leading factor for the use of antibiotics for many doctors. Previous studies have shown that pain in the maxillary sinuses occurs in 80–95% of patients who contact their GP with sinusitis problems [12,23]. Symptoms like pain have been found to be equally prominent for patients both with and without bacterial etiology [12]. Multiple studies have concluded that tension-type facial pain is often misdiagnosed as rhinosinusitis [15,16,24]. The British antimicrobial prescribing guidelines recommend painkillers in the very first line of their guidelines for treatment of acute sinusitis [25]. The Norwegian guidelines for antibiotics do not emphasize treatment of pain as an alternative to antibiotics in their recommendations regarding sinusitis. Painkillers are mentioned as the third point under ‘other treatment’ at the end of the recommendations [7]. Neither the British nor the Norwegian guidelines mention possible other causes of facial pain. This is paradoxical, considering the results of our study. The role of guidelines may also be to guide users to reconsider their diagnosis before prescribing antibiotics, especially in cases like sinusitis where antibiotics should be avoided in most instances.

Conflicting rights and obligations in the physician-patient relationship, as well as the focus on an organization that encourages continuity and professional autonomy, are recurring themes in the literature [26]. These are also important factors for prudent antibiotic prescribing. In addition to focusing on the usefulness of knowing the patient when it comes to diagnostic considerations, several doctors described well-known and challenging patients who often make specific demands regarding antibiotics. Some maintained that you can’t take the fight every time with these patients.

We also found that contextual factors, such as the day of the week, time of day, stress, time pressure, and travel-plans played a role in decision-making regarding treatment. In line with our findings, a study conducted in Australia showed that many GPs prescribe antibiotics for upper respiratory tract infections to meet ‘patient expectations’, due to limited time, poor communication, and diagnostic uncertainty [27]. Some of these factors may be difficult to change as they are more related to the individual human factor and ‘clinical gestalt’. Clinical gestalt refers to the overall clinical impression, which is an intuitive approach to decision making used by physicians in the diagnostic process [28]. In our focus groups, some GPs discussed how patients in severe pain or with demanding situations at home were considered ‘deserving’ of antibiotics. This may indicate that part of the assessment is to evaluate who is more ‘worthy of’ treatment. If patients are better at communicating and ‘pressing the right buttons’ they may be more likely to receive antibiotics.

Many of the doctors in our study were clear about using clinical gestalt and their own experience to make treatment decisions. John Gabbay describes how GPs rely on ‘mindlines’ instead of guidelines [29]. He claims that coffee-room chat may impact evidence-based practice at least as much as all the guidelines that deluge GPs. In our study, several participants held the opinion that phenoxymethylpenicillin did not work for sinusitis. This was explained as something they had experienced, but also as a logical conclusion on their knowledge of sinus anatomy. Our study partially confirms Gabbay’s presentation of a clinical thinking approach based on mindlines may align with the use of guidelines as a tool. Others have also shown that implementing guidelines in general practice is challenging [30,31]. Implementation and quality improvement projects such as audit-and-feedback, group discussions, and academic detailing have significantly reduced antibiotic prescribing for sinusitis, but a disproportionately high prescription rate still exists [4]. To progress, it might be useful to explore other methods of delivering treatment recommendations to GPs.

We have demonstrated that the complex decision-making process makes it challenging to establish a unified treatment strategy for AS among all GPs. In situations where doctors face uncertainty and complex decisions, there has been a growing suggestion of incorporating artificial intelligence (AI) as a decision-making tool. The use of AI to complement GP diagnostic processes has garnered significant interest [32,33]. By being trained with relevant knowledge on diagnoses and decision making, AI has the potential to assist GPs in recognizing and overcoming cognitive biases [32]. Addressing the challenges we identified in our study, AI-based tools could prove valuable in the future, offering reminders for appropriate antibiotic usage and providing recommendations for pain management. Moreover, leveraging advanced language processing technology, these tools can analyze patient records to determine the adherence of antibiotic use to guidelines based on the provided information.

#### Methodological considerations

The lead author, with a background as an ENT specialist, has worked as a municipal superintendent, in the out-of-hours medical service, and as a GP. Part of her background may have influenced her prejudices and understanding of the prerequisites for how a general practitioner works. We were aware of this potential bias during the process. The co- authors are all experienced GPs, providing balance to the discussions. Themes were derived from the data collected and were not identified in advance. The interviews were conducted over two years, in four different regions of Norway, and we interviewed 25 participants with varying ages and experience, giving the study external validity. The research question was focused and well-defined, the relatively homogeneous data had consistent quality, and overall, the researchers were an experienced team. According to Malterud, this points to sufficient information power [21]. We observed cross-sectional agreement in the results, suggesting their potential applicability to other countries with similar primary healthcare systems.

Three of the focus groups were conducted in established CME groups, where participants knew each other beforehand. Previous research supports that such groups provide a safe environment for discussion even when deviating from professional guidelines, hereby strengthening the quality of discussions [34]. We have less knowledge regarding these mechanisms among colleagues at a general practitioner’s office and assume it varies somewhat among offices. Two of the interviews were conducted before the onset of the COVID-19 pandemic, two during the pandemic, and one right after the reopening of Norwegian society. Some participants from the last interviews mentioned that they had not seen sinusitis patients for a long time, as most infected patients were seen by designated infection clinics during the pandemic, potentially influencing their recollection of sinusitis treatment. Therefore, we conducted an additional focus group to ensure we had interviews from before, during and after the pandemic with similar findings.

After analyzing the data, we conducted informant validation, contacting and presenting the main findings to three participants from three different focus groups. All informants acknowledged the findings as reflective of their respective focus group discussion and verified the main results.

The focus groups were conducted in four distinctive regions of Norway, including cities of varying size. Despite all focus groups being city-based, we concluded that we had adequate diversity due to differences in geographical areas and population sizes. We interviewed 5 men and 20 women. The research team discussed this gender bias and the need for additional focus groups. However, we found no apparent gender differences in our findings, and we do not have reason to believe that the treatment of sinusitis is gender sensitive. The participants ranged in age from 27 to 58 years, with an average of 42 years, and had an average of 11.3 years of experience in general practice. This variation in age and experience contributed to rich data.

## Conclusion

GPs found it challenging to identify patients who would benefit from antibiotic treatment for acute sinusitis. Various strategies were used to determine the treatment, with the level of pain being a crucial factor for many GPs. However, the Norwegian guidelines for antibiotics do not emphasize recommending pain treatment for patients with sinusitis when antibiotics are not indicated. Additionally, we found that many doctors lacked confidence in the guidelines and made decisions based on their experience instead. These findings suggest the need for a reassessment of guideline content and its potential communication to GPs in new and innovative ways.
